# Clonal dissemination of the multi-drug resistant *Salmonella enterica *serovar Braenderup, but not the serovar Bareilly, of prevalent serogroup C1 *Salmonella *from Taiwan

**DOI:** 10.1186/1471-2180-9-264

**Published:** 2009-12-17

**Authors:** Chien-Shun Chiou, Jui-Ming Lin, Cheng-Hsun Chiu, Chi-Hong Chu, Shu-Wun Chen, Yung-Fu Chang, Bor-Chun Weng, Jwu-Guh Tsay, Chyi-Liang Chen, Chien-Hsing Liu, Chishih Chu

**Affiliations:** 1 The Central Region Laboratory, Center of Research and Diagnostics, Centers for Disease Control, No. 30, Wenxin S. 3rd Rd., Nantun Dist., Taichung, 40856, Taiwan; 2 Department of Microbiology and Immunology, National Chiayi University, No. 300, University Rd, Chiayi, 60004, Taiwan; 3 Department of Pediatrics, Chang Gung Children's Hospital, 5. Fu-Hsing Street. Kuei-Shan Hsiang, 33375, Taoyuan, Taiwan; 4 Department of Medicine, College of Medicine, Chang Gung University, 259 Wen-Hwa 1st Road, Kwei-Shan, Taoyuan, 33302, Taiwan; 5 Department of Surgery, National Defense Medical Center, No.325, Sec.2, Chenggong Rd., Neihu District, Taipei, 11490, Taiwan; 6 Department of Population Medicine and Diagnostic Sciences, College of Veterinary Medicine, S2-009 Schurman Hall, Box 37, Cornell University, Ithaca, NY 14853-6401, USA; 7 Molecular Infectious Diseases Research Center, Chang Gung Memorial Hospital, 5. Fu-Hsing Street. Kuei-Shan Hsiang, Taoyuan, 33375, Taiwan; 8 Laboratory Department, Tainan Hospital, No. 125, Jhongshan Rd., Tainan City 70043 Taiwan

## Abstract

**Background:**

Nontyphoidal *Salmonella *is the main cause of human salmonellosis. In order to study the prevalent serogroups and serovars of clinical isolates in Taiwan, 8931 *Salmonellae *isolates were collected from 19 medical centers and district hospitals throughout the country from 2004 to 2007. The pulsed-field eletrophoresis types (PFGE) and antibiotic resistance profiles of *Salmonella enterica *serovars Bareilly (*S*. Bareilly) and Braenderup (*S*. Braenderup) were compared, and multi-drug resistance (MDR) plasmids were characterized.

**Results:**

Over 95% of human salmonellosis in Taiwan was caused by five *Salmonella *serogroups: B, C1, C2-C3, D1, and E1. *S*. Typhymurium, *S*. Enteritidis, *S*. Stanley and *S*. Newport were the four most prevalent serovars, accounting for about 64% of isolates. While only one or two major serovars from four of the most prevalent serogroups were represented, four predominant serovars were found in serogroup C1 *Salmonellae*. The prevalence was decreasing for *S*. Choleraeuis and *S*. Braenderup, and S. Virchow and increasing for *S*. Bareilly. *S*. Braenderup mainly caused gastroenteritis in children; in contrast, *S*. Bareiley infected children and elderly people. Both serovars differed by *Xba*I-PFGE patterns. Almost all *S*. Bareilly isolates were susceptible to antibiotics of interest, while all lacked plasmids and belonged to one clone. Two distinct major clones in *S*. Braenderup were cluster A, mainly including MDR isolates with large MDR plasmid from North Taiwan, and cluster B, mainly containing susceptible isolates without R plasmid from South Taiwan. In cluster A, there were two types of conjugative R plasmids with sizes ranging from 75 to 130 kb. Type 1 plasmids consisted of replicons F1A/F1B, *bla*_TEM_, IS*26*, and a class 1 integron with the genes *dfrA12*-*orfF*-*aadA2-qacE*Δ1-*sulI*. Type 2 plasmids belonged to incompatibility group Inc*I*, contained *tnpA*-*bla*_CMY-2_-*blc*-*sugE *genetic structures and lacked both IS*26 *and class 1 integrons. Although type 2 plasmids showed higher conjugation capability, type 1 plasmids were the predominant plasmid.

**Conclusions:**

Serogroups B, C1, C2-C3, D1, and E1 of *Salmonella *caused over 95% of human salmonellosis. Two prevalent serovars within serogroup C1, *S*. Bareilly and cluster B of S. Braenderup, were clonal and drug-susceptible. However, cluster A of *S*. Braenderup was MDR and probably derived from susceptible isolates by acquiring one of two distinct conjugative R plasmids.

## Background

Non-typhoidal *Salmonellae *are major zoonotic pathogens that commonly cause salmonellosis outbreaks. Globally, salmonellosis caused by non-typhoidal salmonellae generally results in about 1.3 billion cases of acute gastroenteritis and 3 million deaths annually [1]. In the United States, *Salmonellae *cause an estimated 1.4 million cases of salmonellosis and over 500 deaths annually [2]. Multi-drug resistant (MDR) *Salmonella*, the global spread of which is mediated by international food trade and travel, is a global public health issue [3,4]. Often, clonal spread of MDR strains has been observed in particular serovars [4-6]. In most instances, resistance genes often associated with integrons and/or transposons are clustered within antimicrobial resistance islands that can be horizontally transferred by conjugative or mobilization plasmids [7].

In serogroup C1, *S*. Bareilly and *S*. Braenderup are closely related according to molecular analysis [8,9]. Both serovars have been highly susceptible to antimicrobials since 1971 [10,11] and are frequently isolated from feces of people with food-borne salmonellosis all over the world [12-16]. However, prevalence of both serovars differs between hosts and regions. In Denmark, *S*. Bareilly was isolated from diverse sources, including humans, animals and animal feed, while *S*. Braenderup was only found in humans [17]. In a study of a broiler-raising plant in the USA, *S*. Bareilly was often found in broilers and finished feed; however, *S*. Braenderup was only observed in hatcheries [18]. In addition, *S*. Braenderup was commonly isolated from cattle and turtles in Sweden [19], pigs [12] and chicken egg shells [20] in USA. These findings imply that animal reservoirs may be important sources of both serovars in human disease.

In this study, prevalent serogroups and serovars were determined for 8,931 *Salmonella *isolates collected from 2004 and 2007 in Taiwan. Because of the genetic similarity between *S*. Bareilly and *S*. Braenderup [8,9], the two serovars were compared with respect to antimicrobial resistance, resistance genes, PFGE and plasmid profiles. Both serovars disseminated clonally and varied in antimicrobial resistance patterns.

## Results

### Prevalent serogroups and serovars

Between 2004 and 2007, over 95% of 8,931 *Salmonella *isolates belonged to serogroups B, C1, C2-C3, D1 and E1 (Table 1). Prevalence differed between serogroups and across time within serogroups: prevalence decreased in serogroups B (46.9%→42.4%) and C1 (14.2%→9.1%) and increased in serogroups C2-C3 (9%→11.3%) and D1 (23.3%→30.2%) over the study period. Such changes were associated with the prevalence of major serovars in each serogroup and were due to only one or two main predominant serovars in each serogroup, except serogroup C1 with four prevalent serovars (Table 1). The top four serovars were *S*. Enteritidis (22.9-28.9%) of serogroup D1, *S*. Typhimurium (20.4-24.7%) and *S*. Stanley (8.2-11.4%) of serogroup B, and *S*. Newport of serogroup C2 (5.6 - 7.3%). In contrast to the decrease in prevalence of *S*. Typhimurium from 2005 to 2007, a gradual increase in prevalence was observed in *S*. Enteritidis.

**Table 1:** Prevalence of *Salmonella *serogroups and their main serovars isolated from human from 2004 to 2007.

Serogroup/Serovar	Number of isolates					**Prevalence (%)**^**2**^				
		
	2004	2005	2006	2007	Total	2004	2005	2006	2007	Total
Serogroup B	1133	1045	938	854	3970	44.3	46.9	44.0	42.4	44.5
*S*. Typhimurium	571	551	441	412	1975	22.3^ab^	24.7^a^	20.7^b^	20.4^b^	22.1^ab^
*S*. Stanley	287	183	242	168	880	11.2	8.2	11.4	8.3	9.9
										
Serogroup C1	364	229	234	184	1101	14.2	10.3	11.0	9.1	11.3
*S*. Choleraesuis	111	65	30	17	223	4.3 (30.5)	2.9 (28.4)	1.41 (12.8)	0.84 (9.23)	2.50 (22.6)
*S*. Braenderup	96	46	66	32	240	3.8 (26.4)	2.1 (20.9)	3.1 (28.2)	1.6 (17.4)	2.7 (23.7)
*S*. Bareilly	54	41	47	54	196	2.1 (14.8)	1.8 (17.9)	2.2 (20.1)	2.7 (29.4)	2.2 (19.4)
*S*. Virchow	43	34	33	19	129	1.7 (11.8)	1.5 (14.8)	1.6 (14.1)	0.9 (10.3)	1.4 (12.8)
Other serovars^1^	60	43	58	62	223	2.3 (16.5)	1.9 (18.8)	2.7 (24.8)	3.1 (33.7)	2.5 (22.1)
										
Serogroup C2-C3	231	246	239	228	944	9.0	11.0	11.2	11.3	10.6
*S*. Newport	144	137	135	147	563	5.6	6.1	6.3	7.3	6.3
*S*. Albany	87	109	104	81	381	3.4	4.9	4.9	4.0	4.3
										
Serogroup D	597	550	583	609	2339	23.3	24.7	27.4	30.2	26.2
*S*. Enteritidis	586	543	567	582	2278	22.9^c^	24.4^bc^	26.6^ab^	28.9^a^	25.5
										
Serogroup E1	122	76	64	70	332	4.8	3.4	3.0	3.5	3.7
*S*. Weltevreden	94	61	556	62	273	3.7	2.7	2.6	3.1	3.1
										
Sum^3^	2447	2147	2058	1954	8736	95.6	96.3	96.6	96.5	96.3
Total *Salmonellae*	2,557	2,228	2,131	2,015	8,931					

^1^Other serogroup C1 serovars include are mainly *S*. Infantis, *S*. Potsdam, *S*. Mbandaka, and *S*. Montevideo.

^2^Numbers in parenthesis indicate the percentage of isolates of a C1 serovar over total serogroup C1 isolates.

^3^Sum is the total number of serogroup B, C1, C2-C3, D, and E isolates.

^abc^Different letters indicate significant difference between years.

### Prevalence of serogroup C1 serovars

*S*. Braenderup, *S*. Choleraesuis, *S*. Bareilly and *S*. Virchow were the predominant serovars in serogroup C1 and consisted of 66 - 84% of total serogroup C1 isolates from 2004 to 2007 (Table 1). Other serovars, including *S*. Infantis, *S*. Potsdam, *S*. Mbandaka, and *S*. Montevideo, were occasionally isolated with prevalence less than 1% for each serovar. Over the study period, the prevalence of *S*. Choleraesuis declined dramatically, and *S*. Braenderup prevalence declined mildly. In contrast, the prevalence of *S*. Bareilly and other serovars gradually increased from 2004 to 2007. Since *S*. Braenderup and *S*. Bareilly were the two main serogroup C1 serovars in 2006-2007 and differed in prevalence trends, 45 *S*. Braenderup and 51 *S*. Bareilly isolates were analyzed for their antimicrobial resistance profiles and genetic characteristics.

### Age distribution of patients

Patients infected with *S*. Braenderup and *S*. Bareilly were separated into four age groups. Although, both serovars were found primarily to infect children (age ≤ 4 years), *S*. Bareilly was isolated far more frequently from the elderly (age ≥ 50 years) (8.9% for *S*. Braenderup vs. 31.4% for *S*. Bareilly, p < 0.05) (Table 2). However, *S*. Braenderup was predominantly isolated from children (68.9% for *S*. Braenderup vs. 49% for *S*. Bareilly, p < 0.05).

**Table 2:** Age prevalence of patient infected by *S*. Bareilly and *S*. Braenderup

	Rate (%) of each age group			
	
Serovar	0 ~ 4	5 ~ 12	13 ~ 50	> 50
*S*. Bareilly	49.0^b^ (25/51)	9.8 (5/51)	9.8 (5/51)	31.4^b^ (16/51)
*S*. Braenderup	68.9^a^ (31/45)	8.9 (4/45)	13.3 (6/45)	8.9^a^ (4/45)

^ab^Different letters indicate significant difference between *S*. Bareilly and *S*. Braenderup (Pp<0.05).

### PFGE phylogenetic analysis

**Figure 1:**
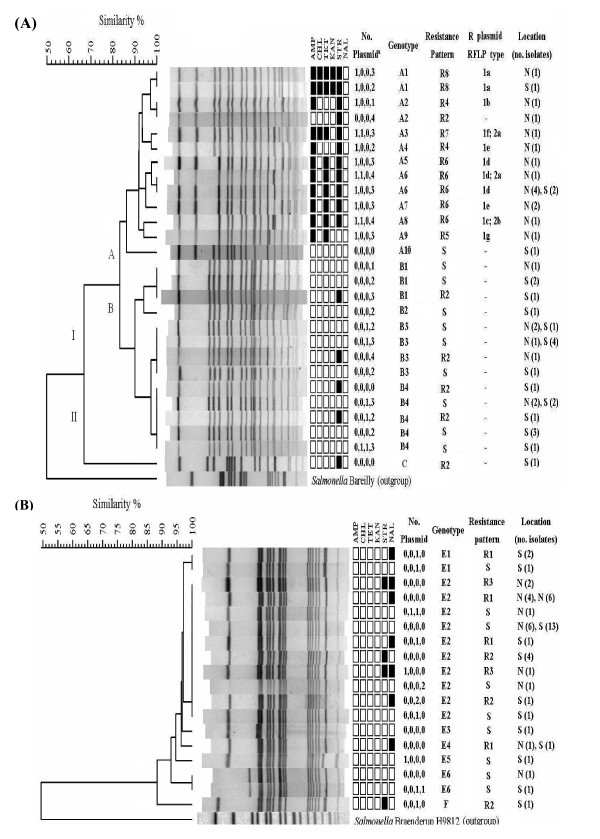
**Dendrograms were constructed by PFGE-*Xba*I patterns to determine the genotypes for *S*. Braenderup (A) and *S*. Bareilly (B) with corresponding information including the number and size of plasmids, PFGE subtypes, antimicrobial resistance patterns and collection location of each isolate**. The dendrograms were generated by the unweighted pair group method with arithmetic mean (UPGMA) using the Dice-predicted similarity value of two patterns. The BioNumerics version 4.5 statistics program was used with settings of 1.0% optimization and 0.7% tolerance. Symbols of black square and white square represent resistant and susceptible respectively. Plasmids were separated into four groups by size. Ex, 1, 1, 1, 3 indicates that this strain harbored 6 plasmids, one is >90 kb, one is from >50 to <90 kb, one is from >6.6 to <50 kb, and three are <6.6 kb.

The clustering analysis of *Xba*I-digested PFGE patterns demonstrated genetic differences between *S*. Braenderup and *S*. Bareilly and within each serovar (Figure 1). In *S*. Braenderup, all isolates were separated into 2 clusters (I and II) at S = 0.68. Most isolates belonged to cluster I, which was further separated into two subgroups (A and B) at S = 0.84 (Figure 1A). In cluster A, 19 isolates were separated into 9 PFGE patterns, and 78.9% (15/19) of the isolates were from northern Taiwan (Figure 1A). In cluster B, 25 isolates were grouped into 4 PFGE patterns, and 72% (18/25) of the isolates were from southern Taiwan (Figure 1A). *S*. Bareilly isolates were highly genetically homogenous and shared more than 90% pattern similarity (Figure 1B).

### Antimicrobial resistance profiles

Among six traditional antibiotics tested, *S*. Braenderup and *S*. Bareilly isolates were almost all susceptible to chloramphenicol (CHL; 6.7% for *S*. Braenderup vs 0% for *S*. Bareilly) and kanamycin (KAN; 4.4% vs 0%) and differed significantly in resistance to ampicillin (AMP, 37.7% for *S*. Braenderup vs 0% for *S*. Bareilly), nalidixic acid (NAL; 0% vs 15.7%), streptomycin (STR, 37.7% vs 15.7%), and tetracycline (TET; 33.3% vs 0%) (Figure 1). Additionally, nine resistance patterns were determined, ranging from susceptibility to all antimicrobials to resistance to four antimicrobials. In *S*. Braenderup, 7 resistance patterns (S, R2, R4 to R8) were found, and significant differences were observed between cluster A (patterns R2, R4-R8) and B (patterns S and R2) for AMP (77.3% vs 0%), STR (63.6% vs 13%) and TET (54.5% vs 13%). In addition, most isolates in cluster A were MDR (73.7%) while most isolates in cluster B were susceptible (84%). In cluster A, pattern R6 (AMP, TET, and STR) was the predominant and was found in four genotypes (A3, A5, A6, and A7). In *S*. Bareilly, most isolates were either susceptible (S pattern; 52.9%) or resistant to one (pattern R1 and R2; 31.4% and 9.8%, respectively) or two (pattern R3; 5.9%) antimicrobials. NAL resistant isolates were found in *S*. Bareilly (patterns R2 and R3) but not in *S*. Braenderup. Since there were susceptible to levofloxacin (LEV) and moxifloxacin (MOX), NAL resistance may result from a mutation in the *gyrA *gene, which encodes a subunit of the enzyme DNA gyrase.

### Characterization of MDR plasmids

**Figure 2:**
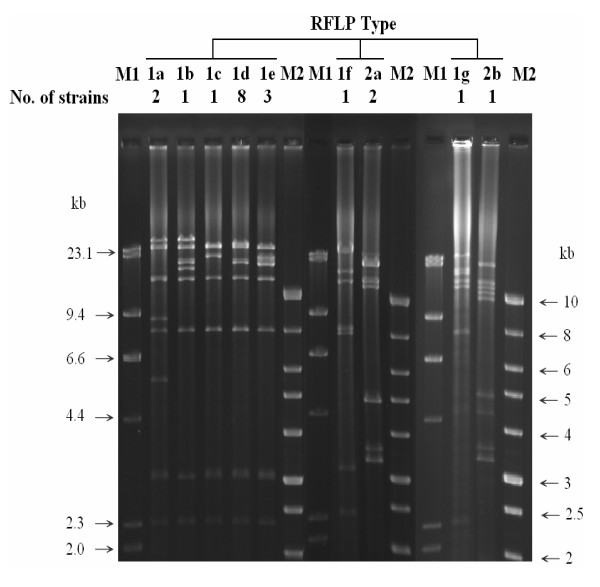
***Hin*dIII-digested RFLP profiles of ampicillin resistance plasmids in *S*. Braenderup isolates**. M1: *Hin*dIII-digested lambda DNA size marker. M2: 1 kb size marker.

**Figure 3:**
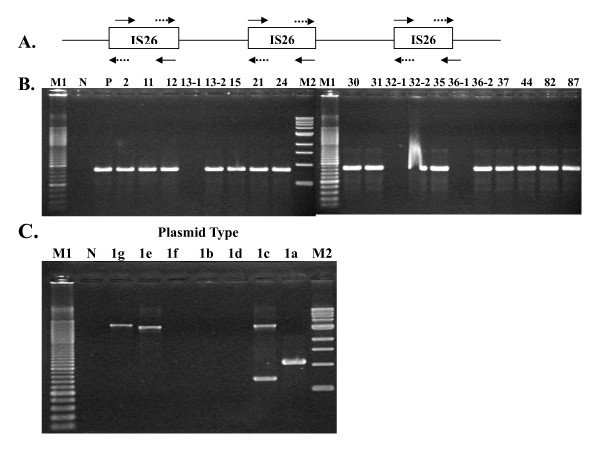
**PCR amplification of IS*26 *and IS*26*-associated DNA fragments**. (A) Primer design. Symbols of arrow and dashed arrow represent IS26in primers and IS26out primers, respectively. (B) PCR products amplified by IS26in primers. (C) PCR products amplified by IS26out primers. M1: 100-bp size marker. N: negative control. M2: 1-kb size marker.

The prevalence of plasmid profile determined by plasmid number and size differed between these two serovars. Most *S*. Braenderup isolates [93.3%, (42/45)] carried plasmids, while few *S*. Bareilly isolates [23.5 % (12/51)] did (Figure 1). Plasmids larger than ca.75 kb were only found in resistance isolates of cluster A with the R4 to R8 patterns. Cluster B *S*. Braenderup isolates and *S*. Bareilly isolates carried smaller plasmids with the size smaller than 6.6 kb or lacked plasmids. Larger plasmids were further identified as R plasmids by analysis of the antimicrobial resistance profiles of *E. coli *pir116 transformants, and assigned to type 1 and 2 based on *Hin*dIII-restriction patterns (Table 3, Figure 2). Further conjugation, antibiotic resistance and PCR characterization of incompatibility and *oriT *types, mobile element IS*26*, class 1 integron, and AMP resistance genes *bla*_TEM _and *bla*_CMY-2 _were performed for these two plasmid types. Type 1 plasmids were separated into 7 subtypes (1a ~1g) based on differences in plasmid size ranging from 99.1 kb to 137.4 kb and restriction pattern. All plasmids carried *bla*_TEM_, replicons F1A and F1B, IS*26*, and a class 1 integron (Additional files 1 and 2: Figure S1 and S2) with a gene cluster of *dfrA12*-*orfF*-*aadA2-qacE*Δ1-*sulI*, conferring resistance to trimethoprim-sulfamethoxazole (Sxt) and disappearing in plasmid 1 g (Table 3), which apparently coincides with that in the plasmid of *S*. Typhimurium (Accession number AB365868). The size of R plasmid was associated with antimicrobial resistance and conjugation capability (Table 3). Only type 1a plasmids, with a size of 137.4 kb and conferring resistance to AMP, CHL, KAN, Sxt and TET, and 1b plasmids, with a size of 122.6 kb and encoding resistance to AMP and Sxt, were capable of conjugation, with efficiencies ranging 4.22 ~ 8.25 × 10^-6^. The other smaller plasmids, with sizes ranging from 99.1 kb to 104.8 kb and encoding resistance to AMP and Sxt for 1c-1e and 1g, and to AMP, CHL, Sxt and TET for 1f, were not capable of conjugation. Due to differences in plasmid size and since IS*26 *could be involved in plasmid transposition and recombination, we performed PCR amplification with the IS26 in primers and IS26out primers for all type 1 plasmids (Figure 3). In contrast to a 1.1-kb PCR product in the largest 1a plasmid, 1b, 1d, and 1e plasmids lacked any PCR products; 1e and 1g plasmids presented 3.1 kb PCR products; and 1c plasmid yielded two PCR products with sizes of 3.1 kb and 0.7 kb. These results suggest that the number of IS*26 *and/or distance between two IS*26 *elements differed among these type 1 plasmids. In contrast to type 1 plasmids, type 2 plasmids were much smaller in size (77.5 kb and 85 kb) and had higher conjugation efficiencies, ranging from 8.41 × 10^-2 ^to 1.28 × 10^-1 ^(Table 3). In addition, type 2 plasmids were the Inc*I1 *plasmid and contained *oriT *as well as *tnpA-bla*_CMY-2_-*blc*-*sugE *(Table 3, Additional files 3: Figure S3).

**Table 3:** Characteristics of MDR plasmids from 17 *S*. Braenderup isolates.

			Antimicrobial resistance gene									
										
Strains	Plasmid RFLP profile	**Antibiogram**^**1**^	*aadA2*	*blaTEM*	*blaCMY-2*	Plasmid size (kb)	Conjugation rate	**Inc**^**3**^	Class I integron	IS*26*	Month of isolation	Number of isolates
*S*. Braenderup 2	1a	ACKTSSxt	+	+	-	137.4	4.22 × 10^-6^	F1A/1B	+	ND	2004.8	2
*E. coli*/p2		ACKSxtT								+		
*S*. Braenderup 96	1a	ACKSSxtT	+	+	-	137.4	6.04 × 10^-6^	F1A/1B	+	ND	2004.8	
*E. coli*/p96		ACKSxtT								+		
*S*. Braenderup 24	1b	ASSxt	+	+	-	122.6	8.25 × 10^-6^	F1A/1B	+	ND	2004.8	1
*E. coli*/p24		ASxt								+		
*S*. Braenderup 87^4^	1d	ASSxtT	+	+	-	102.5	--	F1A/1B	+	ND	2004.7	7
*E. coli*/p30		ASxt								+		
*S*. Braenderup 12	1e	ASSxtT	+	+	-	99.1	-	F1A/1B	+	ND	2005.4	3
*E. coli*/p12		ASxt								+		
*S*. Braenderup 11	1g	ASxtT	-	+	-	104.4	-	F1A/1B	-	ND	2005.1	1
*E. coli*/p11		ASxt								+		
*S*. Braenderup 13		ACSSxtT	+	+	+				+	ND	2004.7	
*E. coli*/p13-1		A	-	-	+	75.5	8.41 × 10^-2^	IncI1	-	-		1
*E. coli*/p13-2	1f	ACSxtT	+	+	-	127.8	-	F1A/1B	+	+		1
*S*. Braenderup 32		ASSxtT	+	+	+				+	ND	2005.9	
*E. coli*/p32-1	2a	A	-	-	+	75.5	8.66 × 10^-2^	IncI1	-	-		1
*E. coli*/p32-2	1d	ASxt	+	+	-	102.5	ND	F1A/1B	+	+		1
*S*. Braenderup 36		ASSxtT	+	+	+				+	ND	2005.5	
*E. coli*/36-1	2b	A	-	-	+	85	1.28 × 10^-1^	IncI1	-	-		1
*E. coli*/p36-2	1c	ASxt	+	+	-	104.8	-	F1A/1B	+	+		1

^1^Abbreviation: A, ampicillin; C, chloramphenicol; K, kanamycin; S, streptomycin; Sxt, trimethoprim-sulfamethoxazole; T, tetracycline.

^2^ND, not determined; +, conjugative; -, .non-conjugative.

^3^Inc, plasmid incompatibility group.

^4^Other 6 isolates 30 from 2005/2, 31 from 2004/10, 35 from 2005/7, 37 from 2005/3, 44 from 2004/6, and 82 from 2004/7 were not tested for conjugation.

^5^Other 2 isolates 15 from 2005/5 and 21 from 2004/9 were not tested for conjugation.

## Discussion

Human salmonellosis was limited to five *Salmonella *serogroups: B, C1, C2-C3, D1, and E1 (Table 1). Despite the decrease in prevalence of *S*. Typhimurium and the increase in the prevalence of *S*. Enteritidis from 2005 to 2007, serogroups B and D *Salmonellae *were the major pathogens for foodborne salmonellosis in Taiwan due to *S*. Typhimurium, *S*. Stanley, and *S*. Enteritidis of serogroup D1 being the three most prevalent serovars overall. Although the prevalence of serogroups C1 and C2-C3 were similar (around 11%), 4 prevalent serovars and 2 main serovars were found in serogroup C1 and serogroup C2-C3, respectively. In the present study, a shift in prevalence was observed in these four prevalent serogroup C1 serovars: a rapidly decrease in the prevalence of *S*. Choleresuis, mainly due to enhancement of sanitation and control of swine in Taiwan, and an increase in prevalence of *S*. Bareilly and other serovars (Table 1). Compared to the 1.6% increase in the prevalence of *S*. Braenderup from 1978 to 1987 in southern Taiwan [21], the change in the prevalence of isolates in this study ranged from 1.6% to 3.8%, with a trend of decrease from 2004 to 2007, except an increase of *S*. Braenderup infection in 2006 (Table 1), suggesting possibly occurrence of outbreaks in this year.

Contrary to earlier reports that *S*. Bareilly and *S*. Braenderup are closely related genetically [8,9], resistant to 10 *Salmonella *bacteriophages [22], and infect immuno-compromised patients, differences between *S*. Braenderup and *S*. Bareilly were found in the prevalence trend from 2004 to 2007 (Table 1), patients' age group (Table 2), and plasmid profile as well as antimicrobial resistance groups and *Xba*I-PFGE patterns (Figure 1A). In addition to genetic differences between these two serovars, differences in animal hosts were also observed in both serovars based on the geographic regions from which they were isolated [13,17,18,23]. In this study, we found that *S*. Bareilley isolates were highly homogeneous genetically and that *S*. Braenderup isolates were much diverse in our PFGE and plasmid analysis (Figure 1). This may explain why *S*. Braenderup, but not *S*. Bareilly, has been frequently reported [19,20,24]. To differentiate *S*. Braenderup, several molecular methods have been developed, including phage typing [25] and plasmid analysis as performed in this study (Table 1, Figure 1 and 2).

Unlike MDR *S*. Choleraesuis isolated from pigs and humans [5,6], *S*. Braenderup and *S*. Bareilly isolated from pigs were highly susceptible to antibiotics in 1971 [10]. In addition, in a study of resistance to 11 antibiotics for *Salmonella *isolated from turtles, *S*. Bareilly was still susceptible to all antibiotics, and, in contrast, few *S*. Braenderup isolates were resistant to gentamycin (6/15), sulfisoxazole (6/15) and TET (2/15) [11]. In our study, almost all of the cluster A isolates of *S*. Braenderup were MDR and associated with large MDR plasmids (Table 3, Figure 1). Although RFLP analysis separated type 1 plasmids into 7 subtypes, based on antimicrobial resistance encoded by these plasmids, 3 subtypes were observed, conferring resistance to AMP and Sxt (1b-1e and 1g), AMP, CHL, Sxt, and TET (1f) and AMP, CHL, KAN, Sxt and TET (1a), respectively (Table 3). Apparently, the *dfrA12*-*orfF*-*aadA2-qacE*Δ1-*sulI *region of class 1 integrons, which is frequently found in MDR *Salmonella *[26-28], was located on MDR plasmid and conferred resistance to Sxt (Table 3). Insertion sequence IS*26 *existed in all (Table 3) and differed from plasmids in *S*. Braenderburg found in Spain [29]. The size change in type 1 plasmids may be due to presence of multiple IS*26 *elements that may be involved in plasmid rearrangement (Figure 3).

Although conjugation capability of type 2 plasmids was higher than that of type 1 plasmids, we only identified coexistence of type 1 and 2 plasmids in three *S*. Braenderup isolates, which differed in isolation day and PFGE pattern (Table 3). Isolate 13 with type 1f and 2a plasmids was collected in July of 2004 from Taipei. Isolate 32 with type 1d and 2a plasmids and isolate 36 with 1c and 2b plasmids were collected in March and May of 2005, respectively, from Taichung (Table 3). Only one isolate 44 with a type 1d plasmid was collected before those three isolates, in June of 2004 from Taichung. These results suggest possibly that isolate 32 with A6 genotype and R6 resistance pattern may be derived from isolate 44 with a type 1 plasmid, A4 genotype and R6 resistance pattern by introduction of a type 2 plasmid. Interestingly, type 2 plasmids are Inc*I1 *plasmids, carrying the *tnpA*-*bla*_CMY-2_-*blc*-*sugE *structure (Table 3). AmpC β-lactamases are broadly distributed among the *Enteribacteriaceae*, and plasmid-mediated AmpC β-lactamases include ACC, ACT, CFE, CMY, DHA, FOX, LAT, MIR, and MOX [30]. At least three transposase associated genetic structures for *bla*_CMY _include IS*Ecp1-bla*_CMY-2_-*blc-sugE, ISCR1-bla*_CMY-9_-*yqgF-yqgE *and *IS26-frdC-frdD-ampR-bla*_*CMY-13*_-*blc-sugE-IS26 *[30]. Recently, *bla*_CMY _has been shown to be broadly spread in *Salmonella *worldwide [29,31,32] and to be present in *S*. Braenderup [33]. In Taiwan, since we reported the *tnpA*-*bla*_CMY-2_-*blc*-*sugE *structure in *S*. Choleraesuis in 2004 [34], this transposon-like element has been found in other *Salmonella *serovars and *Enterobacteriaceae *[32]. In the present study, we first reported that *S*. Braenderup harbors *tnpA*-*bla*_CMY-2_-*blc*-*sugE *on a type 2 plasmid. Comparing this plasmid with the 138-kb plasmid pSC138 (accession no. NC_006856) of *S*. Choleraesuis, both are Inc*I1 *plasmids with the *tnpA*-*bla*_CMY-2_-*blc*-*sugE *structure. However, type 2 plasmids were conjugative and much smaller in size due to lack of a 60-kb DNA region with multiple integrons and transposons, which carry MDR genes [35-37].

## Conclusions

Over 95% cases of human salmonellosis surveyed in this study were caused by 5 *Salmonella *serogroups: B, C1, C2, D1, and E1. As two prevalent serogroup C1 serovars, *S*. Braenderup and S. Bareiley differed in patients' age groups and *Xba*I-PFGE patterns. Both serovars were clonally disseminated and drug-susceptible. However, in *S*. Braenderup, cluster A MDR isolates were derived from susceptible isolates by sequential introduction of two distinct R plasmids. Type 1 plasmids carry *bla*_TEM_, F1A/F1B replicons, insertion sequence IS*26*, and a class 1 integron with a gene cluster comprised of *dfrA12*-*orfF*-*aadA2-qacE*Δ1-*sulI*. In contrast, type 2 plasmids consist of Inc*I1 *replicon and *tnpA*-*bla*_CMY-2_-*blc*-*sugE*. Although type 2 plasmids showed higher conjugation capability, type 1 plasmids were the predominant plasmids responsible for MDR dissemination in *S*. Braenderup.

## Methods

### Bacterial isolates

*Salmonella *isolates were collected from 19 medical centers and district hospitals located throughout Taiwan from 2004 to 2007. Serotypes of the isolates were determined in the *Salmonella *Reference Laboratory of Centers for Disease Control (CDC), Department of Health, Taiwan, with antisera purchased from S&A Reagents Lab (Bangkok, Thailand), Denka Seiken (Tokyo, Japan), Statens Serum Institut (Copenhagen, Denmark), and a local biotech company, LTK Biolaboratories (Taoyuan, Taiwan). Phase induction was performed using a paper-bridged method developed by the Taiwan CDC [38]. In total, 51 *S*. Bareilly isolates and 45 *S*. Braenderup isolates collected in 2004 and 2005 were selected for further characterization. Isolates were separated into two groups based on their geographic origin: the north Taiwan group, consisting of isolates collected from north of Taichung county (including Taichung county), and the south Taiwan group, consisting of isolates collected from south of Taichung county.

### Antimicrobial susceptibility testing

Antimicrobial susceptibility testing was performed using the disc diffusion method in accordance with the guidelines of the CLSI standards [39] with 7 antibiotics: ampicillin (AMP, 50 μg), chloramphenicol (CHL, 20 μg), kanamycin (KAN, 30 μg), streptomycin (STR, 10 μg), tetracycline (TET, 12 μg), trimethoprim-sulfamethoxazole (Sxt, 23.75/1.25 μg), and quinolone antibiotics including nalidixic acid (NAL, 30 μg), levofloxacin (LEV, 5 μg) and moxifloxacin (MOX, 5 μg). The antimicrobials were purchased from BD (Becton Dickinson and Company, Sparks, Maryland, USA). *Escherichia coli *ATCC 25922 was used as the reference strain. An MDR isolate was defined as having resistance to three or more antibiotics belonging to different antibiotic classes.

### Pulsed-field gel electrophoresis (PFGE)

The PulseNet Standardized Laboratory PFGE Protocol for Molecular Subtyping of *Echerichia coli *O157:H7, non-typhoidal *Salmonella *serotypes, and *Shigella sonnei *[40] was used for analysis of the *Salmonella *isolates: 10 U of *Xba*I were used for the restriction digestion. PFGE images were analyzed by using the fingerprint analysis software BioNumerics version 4.5 (Applied Maths). A unique PFGE pattern was defined as one or two DNA bands differing between PFGE patterns of two isolates. A dendrogram was generated by the unweighted pairgroup method with arithmetic mean (UPGMA) algorithm using the Dice-predicted similarity value of two *Xba*l-digested PFGE patterns.

### Plasmid profile analysis

Plasmid profiles of each isolate were determined by the Kado and Liu method [41], and plasmid size was estimated by comparison with the plasmids of two *S*. Choleraesuis strains: OU7085 (50 kb and 6.6 kb) and OU7526 (50 kb and 90 kb).

### Restriction fragment length polymorphism (RFLP) and antibiotic susceptibility analysis of the MDR-plasmid

Large plasmids (> 50 kb) of 17 AMP and STR-resistant *S*. Braenderup isolates were characterized. Plasmid DNA was purified from resistant wild-type isolates by the alkaline lysis method [42] and then transformed into the competent *E. coli *strain pir116 (STR^R^), which was prepared by the CaCl_2 _method. Transformants were selectively grown on LB agar plates supplemented with AMP (100 μg/ml) and further tested for resistance to CHL, TET, and KAN, but not for resistance to STR, since the recipient strain was inherently resistant to streptomycin. The antibiotic resistance genes *bla*_TEM_, *aadA*, and *bla*_CMY-2_, class 1 integron as well as the insertion sequence IS*26 *and its related DNA fragments were amplified using the primers listed in Table 4. The genes *bla*_SHV _and *bla*_CTX-M3 and M14 _were also detected by the multiplex method [43]. The R-plasmids of each transformant were purified by use of the Geneaid Plasmid Midi Kit (Geneaid, Taiwan) and were digested with *Hin*dIII (New England Biolabs, USA) to determine similarity. Plasmid DNA fragments were separated by electrophoresis through a 0.6 % SeaKem GTG agarose gel (Cambrex Bio Science Rockland, Inc., Rockland, ME, USA) at 25 V for 16 h. The PCR product of class 1 integron was purified by DNA Clean/Extraction kit (GeneMark, Taiwan) and sequenced by Mission Biotech co. (Taiwan).

**Table 4:** The PCR primers for PCR and size of PCR products

Primer	Target	DNA sequence (5' to 3')	Product Sizesize	Note
Tem-F	*bla*_TEM_	GAAGATCAGTTGGGTGCACGAGT	550 bp	This study
Tem-R		CAACTTTATCCGCCTCCATCCAGT		
STR-F1	*aadA2*	AGACGCTCCGCGCTATAGAAGT	203 bp	(46)
STR-R1		CGGACCTACCAAGGCAACGCT		
CS-F	*CS region*	GGCATCCAAGCAGCAAG	Variable	(47)
CS-R		AAGCAGACTTGACCTGA		
1.9CS-F	*Flanking region of CS region*	CTGCTGCGTAACATCGTTGCT	Variable	This study
1.9CS-R		GGCGAGATCATCAAGTCAGT		
ColE1-F	ColE1 *ori*T	CAAATGCTGTCCTTCCAGTGT	225 bp	This study
ColE1-R		CTCAGTTCGGTGTAGGTCGT		
F-F	Inc*FI ori*T	CAACAACGCGCCGACACCGT	288 bp	This study
F-R		CCCTTCCTGTCGACGCTTCT		
R100-F	Inc*F2 ori*T	CCACCAAAAGCACCACACACT	266 bp	This study
R100-R		AGACACTCCTAGCAGCGCCT		
pSC138-F	Inc*I ori*T	TGTCACGAACATCTGCCAGT	193 bp	This study
pSC138-R		GAGAGAAAGTGCCCATGGCT		
IS26in-F	*IS*26	GGCACTGTTGCAAAGTTAGC	820 bp	DQ390455.1
IS26in-R		GGCACTGTTGCAAATAGTCG		
IS26out-F	Variable	GCTAACTTTGCAACAGTGCC	Variable	DQ390455.1
IS26out-R		CGACTATTTGCAACAGTGCC		
Tn-F	Tn	ACCTAGATTCTACGTCAGTAC	Variable	(35)
AmpC-F	AmpC	CAAGTTTGATTCCTTGGACTCT		AY253913
AmpC-R		CTCATCGTCAGTTATTGCAGCT		
SugE-R	*sugE*	GCCTGATATGTCCTGGATCGT		

### Plasmid conjugation and incompatibility group

Transferability of R plasmids from each RFLP group was determined by performing the conjugation test following a previously described method [44] with NAL-resistant *S*. Typhimurium LBNP4417 as the recipient strain. Briefly, 0.6 ml of overnight culture of donor strain was mixed with 1 ml of the overnight recipient strain. Then 1 ml fresh LB broth was added, and the mixture was incubated at 37°C with shaking at 100 rpm for 4 h. The bacterial solution was diluted at 10^1^, 10^3^, and 10^5 ^times with LB broth, and then 100 μl of the diluted solution was plated on MacConkey agar supplemented with AMP (100 μg/ml) and/or NAL (15 μg/ml). Conjugation efficiency was calculated by determining the number of transconjugants relative to the total number of recipients. Four primer sets were used to amplify the *oriT *regions of the ColE1, F (IncFI), R100 (IncFII), and pSC138 (Inc*I1*-like) plasmids (Table 1). In addition, replicon types of these resistant plasmids were determined as described by Carattoli et al. [45].

### Statistical analysis

The difference in the antimicrobial resistance rates between two serovars was analyzed by the independent t test. P values of < 0.05 were considered significant.

## List of abbreviations

AMP: ampicillin; CDC: Center for Disease Control; CHL: cloramphenicol; KAN: kanamycin; LEV: levofloxacin; MDR: multi-drug resistance; MOX: moxifloxacin; NAL: nalidixic acid; PCR: polymerase chain reaction; PFGE: pulsed-field gel electrophoresis; QRDR: quinolone resistance determining region; RFLP: restriction fragment length polymorphism; STR: streptomycin; Sxt: trimethoprim-sulfamethoxazole; TET: tetramycin; UPGMA: unweighted pairgroup method with arithmetic mean.

## Authors' contributions

CC designed, instructed and supervised most aspects of this project. CSC did PFGE analysis and prepared the manuscript. JML and SWC performed the experiments and data analysis. CHC, BCW and JGT assisted in the design of the study and helped to prepare the manuscript. CLC, CHC, and CHL gave useful comments and critically read the manuscript. YFC edited and revised the manuscript. All authors read and approved the final manuscript.

## Authors' information

Chien-Shun Chiou is Chief Investigator of The Central Region Laboratory, Center of Research and Diagnostics, Centers for Disease Control, Taichung, Taiwan. Jui-Ming Lin and Shu-Wun Chen are research assistants, Bor-Chun Weng is an assistant professor, Jwu-Guh Tsay is a professor, and Chishih Chu is the chairman of Department of Microbiology and Immunology, National Chiayi University, Chiayi, Taiwan. Cheng-Hsun Chiu is a professor in the Department of Pediatrics, Chang Gung Children's Hospital and Chang Gung University College of Medicine, Taoyuan, Taiwan, Chi-Hong Chu is the superintendent of the National Defense Medical Center, Taipei, Taiwan. Yung-Fu Chang is a professor in the Department of Population Medicine and Diagnostic Sciences, College of Veterinary Medicine, Cornell University, Ithaca, NY 14853, USA. Chyi-Liang Chen is an assistant professor at the Molecular Infectious Diseases Research Center, Chang Gung Memorial Hospital, Taoyuan, Taiwan. Chien-Hsing Liu is the director of the Laboratory Department, Tainan Hospital, Taiwan, ROC.

## Supplementary Material

Additional file 1 **Electrophoretic pattern of 1.9 kb PCR products of CS region amplified from type 1 plasmids**. All type 1 plasmids consisted of CS region, except type 1 g and 2 plasmids. Click here for file

Additional file 2 **Electrophoretic profile of inverted PCR products of CS-flanking region amplified from type 1 plasmids**. Inversed PCR of CS flanking region amplified same PCR products from all type 1 plasmids, except those plasmid that did not show any PCR product of CS region. Click here for file

Additional file 3 **PCR amplification of plasmid-mediated *tnpA-bla*_CMY-*2*_*-blc-sugE *genetic structure of type 2 plasmids**. All type 2 plasmids consisted of *tnpA-bla*_CMY-*2*_*-blc-sugE *genetic structure. Click here for file

## References

[B1] Pang T Bhutta ZA Finlay BB Altwegg M Typhoid fever and other salmonellosis: a continuing challenge Trends Microbiol 1995 3 253 255 10.1016/S0966-842X(00)88937-4 7551636

[B2] Mead PS Slutsker L Dietz V McCaig LF Bresee JS Shapiro C Griffin PM Tauxe RV Food-related illness and death in the United States Emerg Infect Dis 1999 5 607 625 10.3201/eid0505.990502 10511517 PMC2627714

[B3] Aarestrup FM Hendriksen RS Lockett J Gay K Teates K McDermott PF White DG Hasman H Sorensen G Bangtrakulnonth A Pornreongwong S Pulsrikarn C Angulo FJ Gerner-Smidt P International spread of multidrug-resistant *Salmonella *Schwarzengrund in food products Emerg Infect Dis 2007 13 726 731 17553251 10.3201/eid1305.061489 PMC2738437

[B4] Butaye P Michael GB Schwarz S Barrett TJ Brisabois A White DG The clonal spread of multidrug-resistant non-typhi *Salmonella *serotypes Microbes Infect 2006 8 1891 1897 10.1016/j.micinf.2005.12.020 16714135

[B5] Chang CC Lin YH Chang CF Yeh KS Chiu CH Chu C Chien MS Hsu YM Tsai LS Chiou CS Epidemiologic relationship between fluoroquinolone-resistant *Salmonella enterica *Serovar Choleraesuis strains isolated from humans and pigs in Taiwan (1997 to 2002) J Clin Microbiol 2005 43 2798 2804 10.1128/JCM.43.6.2798-2804.2005 15956400 PMC1151913

[B6] Chiu CH Su LH Chu C Chia JH Wu TL Lin TY Lee YS Ou JT The emergence in Taiwan of fluoroquinolone resistance in *Salmonella enterica *serotype Choleraesuis N Engl J Med 2002 346 413 419 10.1056/NEJMoa012261 11832529

[B7] Miriagou V Carattoli A Fanning S Antimicrobial resistance islands: resistance gene clusters in *Salmonella *chromosome and plasmids Microbes Infect 2006 8 1923 1930 10.1016/j.micinf.2005.12.027 16716633

[B8] Rasschaert G Houf K Imberechts H Grijspeerdt K De Zutter L Heyndrickx M Comparison of five repetitive-sequence-based PCR typing methods for molecular discrimination of *Salmonellaenterica *isolates J Clin Microbiol 2005 43 3615 3623 10.1128/JCM.43.8.3615-3623.2005 16081887 PMC1234001

[B9] Kim HJ Park SH Kim HY Comparison of *Salmonella enterica *Serovar Typhimurium LT2 and non-LT2 *Salmonella *genomic sequences, and genotyping of *Salmonellae *by using PCR Appl Environ Microbio 2006 72 6142 6151 10.1128/AEM.00138-06 PMC1563604 16957240

[B10] Pocurull DW Gaines SA Mercer HD Survey of infectious multiple drug resistance among *Salmonella *isolated from animals in the United States Appl Microbiol 1971 21 358 362 10.1128/am.21.2.358-362.1971 PMC377176 5205089

[B11] Poppe C Kolar JJ Demczuk WH Harris JE Drug resistance and biochemical characteristics of *Salmonella *from turkeys Can J Vet Res 1995 59 241 248 8548684 PMC1263777

[B12] Centers for Disease Control and Prevention (CDC) PHLIS *Salmonella *Surveillance Annual Summary, 2005. US Department of Health and Human Services, CDC 2007

[B13] Martin WJ Ewing WH Prevalence of Serotypes of *Salmonella* Appl Microbiol 1969 17 111 117 5774752 10.1128/am.17.1.111-117.1969 PMC377623

[B14] de Jong B Oberg J Svenungsson B Outbreak of salmonellosis in a restaurant in Stockholm, Sweden, September - October 2006 Euro Surveill 2007 12 E13 14 18005653 10.2807/esm.12.11.00749-en

[B15] Gupta SK Nalluswami K Snider C Perch M Balasegaram M Burmeister D Lockett J Sandt C Hoekstra RM Montgomery S Outbreak of *Salmonella *Braenderup infections associated with Roma tomatoes, northeastern United States, 2004: a useful method for subtyping exposures in field investigations Epidemiol Infect 2007 135 1165 1173 10.1017/S0950268807007911 17274858 PMC2870677

[B16] Urfer E Rossier P Mean F Krending MJ Burnens A Bille J Francioli P Zwahlen A Outbreak of *Salmonella *Braenderup gastroenteritis due to contaminated meat pies: clinical and molecular epidemiology Clin Microbiol Infect 2000 6 536 542 10.1046/j.1469-0691.2000.00148.x 11168047

[B17] Grunnet K Nielsen B *Salmonella *Types Isolated from the Gulf of Aarhus Compared with Types from Infected Human Beings, Animals, and Feed Products in Denmark Appl Microbiol 1969 18 985 990 4905700 10.1128/am.18.6.985-990.1969 PMC378180

[B18] Kaufmann AF Feeley JC Culture survey of *Salmonella *at a broiler-raising plant Public Health Rep 1968 83 417 422 4967445 PMC1891059

[B19] Boqvist S Hansson I Bjerselius UN Hamilton C Wahlström H Noll B Tysen E Engvall A *Salmonella *Isolated from Animals and Feed Production in Sweden Between 1993 and 1997 Acta Vet Scand 2003 44 181 197 10.1186/1751-0147-44-181 15074631 PMC1831546

[B20] Ching-Lee MR Katz AR Sasaki DM Minette HP *Salmonella *egg survey in Hawaii: evidence for routine bacterial surveillance Am J Public Health 1991 81 764 766 10.2105/AJPH.81.6.764 2029051 PMC1405151

[B21] Peng CF Incidence and antimicrobial resistance of *Salmonella *serotypes in southern Taiwan from 1978 through 1987 Gaoxiong Yi Xue Ke Xue Za Zhi 1992 8 247 54 1619701

[B22] Atterbury RJ Van Bergen MAP Ortiz F Lovell MA Harris JA De Boer A Wagenaar JA Allen VM Barrow PA Bacteriophage Therapy To Reduce *Salmonella *Colonization of Broiler Chickens Appl Environ Microbiol 2007 73 4543 4549 10.1128/AEM.00049-07 17526794 PMC1932804

[B23] Langeland G *Salmonella *spp. in the working environment of sewage treatment plants in Oslo, Norway Appl Environ Microbiol 1982 43 1111 1115 7103478 10.1128/aem.43.5.1111-1115.1982 PMC244194

[B24] Savage W Problems of *Salmonella *Food-poisoning Br Med J 1956 2 317 323 10.1136/bmj.2.4988.317 PMC2035108 13342478

[B25] Sechter I Gerichter CB Phage Typing Scheme for *Salmonella braenderup* Appl Microbiol 1968 16 1708 1712 5726147 10.1128/am.16.11.1708-1712.1968 PMC547744

[B26] Antunes P Machado J Sousa JC Peixe L Dissemination amongst humans and food products of animal origin of a *Salmonella *Typhimurium clone expressing an integron-borne OXA-30 beta-lactamase J Antimicrob Chemother 2004 54 429 34 10.1093/jac/dkh333 15243023

[B27] Hsu SC Chiu TH Pang JC Hsuan-Yuan CH Chang GN Tsen HY Characterisation of antimicrobial resistance patterns and class 1 integrons among *Escherichia coli *and *Salmonella enterica *serovar Choleraesuis strains isolated from humans and swine in Taiwan Int J Antimicrob Agents 2006 27 383 391 10.1016/j.ijantimicag.2005.11.020 16621462

[B28] Molla B Miko A Pries K Hildebrandt G Kleer J Schroeter A Helmuth R Class 1 integrons and resistance gene cassettes among multidrug resistant *Salmonella *serovars isolated from slaughter animals and foods of animal origin in Ethiopia Acta Trop 2007 103 142 149 10.1016/j.actatropica.2007.05.018 17658448

[B29] Martínez N Mendoza MC Rodríguez I Soto S Bances M Rodicio MR Martínez N Detailed structure of integrons and transposons carried by large conjugative plasmids responsible for multidrug resistance in diverse genomic types of *Salmonella enterica *serovar Brandenburg J Antimicrob Chemothe 2007 60 1227 1234 10.1093/jac/dkm336 17827139

[B30] Jacoby GA AmpC beta-lactamases Clin Microbiol Rev 2009 22 161 182 10.1128/CMR.00036-08 19136439 PMC2620637

[B31] Arlet G Barrett TJ Butaye P Cloeckaert A Mulvey MR White DG *Salmonella *resistant to extended-spectrum cephalosporins: prevalence and epidemiology Microbes Infect 2006 8 1945 1954 10.1016/j.micinf.2005.12.029 16714134

[B32] Su LH Chen HL Chia JH Liu SY Chu C Wu TL Chiu CH Distribution of a transposon-like element carrying *bla *(CMY-2) among *Salmonella *and other *Enterobacteriaceae* J Antimicrob Chemother 2006 57 424 9 10.1093/jac/dki478 16396917

[B33] Gray JT Hungerford LL Fedorka-Cray PJ Headrick ML Extended-Spectrum-Cephalosporin Resistance in *Salmonella enterica *Isolates of Animal Origin Antimicrob Agents Chemother 2004 48 3179 3181 10.1128/AAC.48.8.3179-3181.2004 15273145 PMC478523

[B34] Chiu CH Su LH Chu C Chia JH Wu TL Lin TY Lee YS Ou JT Isolation of *Salmonella enterica *serotype choleraesuis resistant to ceftriaxone and ciprofloxacin Lancet 2004 363 1285 126 10.1016/S0140-6736(04)16003-0 15094275

[B35] Chiou CS Jones AL Nucleotide sequence analysis of a transposon (Tn*5393*) carrying streptomycin resistance genes in Erwinia amylovora and other gram-negative bacteria J Bacteriol 1993 175 732 40 8380801 10.1128/jb.175.3.732-740.1993 PMC196212

[B36] Pasquali F Kehrenberg C Manfreda G Schwarz S Physical linkage of Tn*3 *and part of Tn*1721 *in a tetracycline and ampicillin resistance plasmid from *Salmonella *Typhimurium J Antimicrob Chemother 2005 55 562 5 10.1093/jac/dkh553 15731203

[B37] Rao S Maddox CW Hoien-Dalen P Lanka S Weigel RM Diagnostic accuracy of class 1 integron PCR method in detection of antibiotic resistance in *Salmonella *isolates from swine production systems J Clin Microbiol 2008 46 916 920 10.1128/JCM.01597-07 18174294 PMC2268369

[B38] Chiou CS Huang JF Tsai LH Hsu KM Liao CS Chang HL A simple and low-cost paper-bridged method for *Salmonella *phase reversal Diagn Microbiol Infect Dis 2006 54 315 317 10.1016/j.diagmicrobio.2005.10.009 16466895

[B39] Clinical and Laboratory Standards Institute. M100-S17 Performance standards for antimicrobial susceptibility testing; 16th informational supplement 2007 Clinical and Laboratory Standards Institute, Wayne, PA

[B40] Ribot EM Fair MA Gautom R Cameron DN Hunter SB Swaminathan B Barrett TJ Standardization of pulsed-field gel electrophoresis protocols for the subtyping of *Escherichia coli *O157:H7, *Salmonella*, and *Shigella *for PulseNet Foodborne Pathog Dis 2006 3 59 67 10.1089/fpd.2006.3.59 16602980

[B41] Kado CI Liu ST Rapid procedure for detection and isolation of large and small plasmids J Bacteriol 1981 145 1365 1373 7009583 10.1128/jb.145.3.1365-1373.1981 PMC217141

[B42] Birnboim HC Doly J rapid alkaline extraction procedure for screening recombinant plasmid DNA Nucleic Acids Res 1979 7 1513 1523 10.1093/nar/7.6.1513 388356 PMC342324

[B43] Chia JH Chu C Su LH Chiu CH Kuo AJ Sun CF Wu TL Development of a multiplex PCR and SHV melting-curve mutation detection system for detection of some SHV and CTX-M beta-lactamases of *Escherichia coli*, *Klebsiella pneumoniae*, and *Enterobacter cloacae *in Taiwan J Clin Microbiol 2005 43 4486 4491 10.1128/JCM.43.9.4486-4491.2005 16145096 PMC1234143

[B44] Chu C Chiu CH Chu CH Ou JT Nucleotide and amino acid sequences of oriT-traM-traJ-traY-traA-traL regions and mobilization of virulence plasmids of *Salmonella enterica *serovars *enteritidis, gallinarum-pullorum, and typhimurium* J Bacteriol 2002 184 2857 2862 10.1128/JB.184.11.2857-2862.2002 12003924 PMC135071

[B45] Carattoli A Bertini A Villa L Falbo V Hopkins KL Threlfall EJ Identification of plasmids by PCR-based replicon typing J Microbiol Methods 2005 63 219 228 10.1016/j.mimet.2005.03.018 15935499

