# Supplementary material for: Clonal dissemination of the multi-drug resistant Salmonella enterica serovar Braenderup, but not the serovar Bareilly, of prevalent serogroup C1 Salmonella from Taiwan
Source: BMC Microbiol. 2009 Dec 17;9:264. doi: 10.1186/1471-2180-9-264 (PMC2806260; doi:10.1186/1471-2180-9-264)
Supplement: Additional file 1 — Electrophoretic pattern of 1.9 kb PCR products of CS region amplified from type 1 plasmids. All type 1 plasmids consisted of CS region, except type 1 g and 2 plasmids. [file 1471-2180-9-264-S1.PDF]

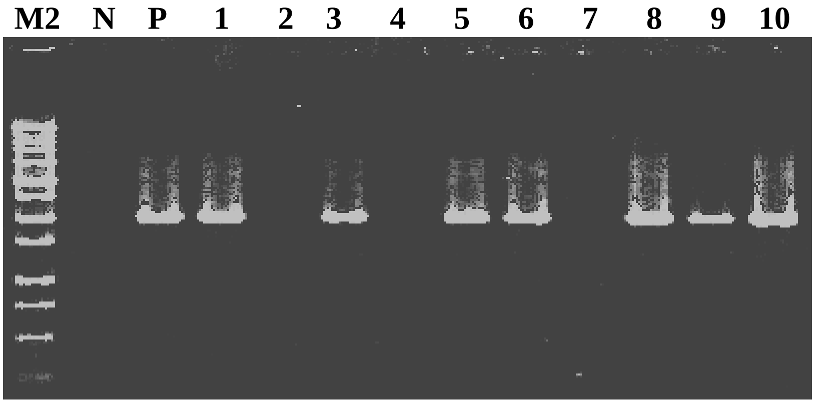

Figure S1. Electrophoretic pattern of 1.9 kb PCR products of CS region amplified from plasmids: p2 (lane 1), p11 (lane 2), p12 (lane 3), p13-1 (lane 4), p13-2 (lane 5), p15 (lane 6), p36-1 (lane 7), p21 (lane 8), p24 (lane 9), p30 (lane 10). M1: 100 bp size marker; N: negative control without any DNA template; P: positive control with pSC138. PCR products were separated by 1.0% agarose for 1.5 hr at 50 V and visualized under UV illumination.
