# Supplementary material for: Clonal dissemination of the multi-drug resistant Salmonella enterica serovar Braenderup, but not the serovar Bareilly, of prevalent serogroup C1 Salmonella from Taiwan
Source: BMC Microbiol. 2009 Dec 17;9:264. doi: 10.1186/1471-2180-9-264 (PMC2806260; doi:10.1186/1471-2180-9-264)
Supplement: Additional file 2 — Electrophoretic profile of inverted PCR products of CS-flanking region amplified from type 1 plasmids. Inversed PCR of CS flanking region amplified same PCR products from all type 1 plasmids, except those plasmid that did not show any PCR product of CS region. [file 1471-2180-9-264-S2.PDF]

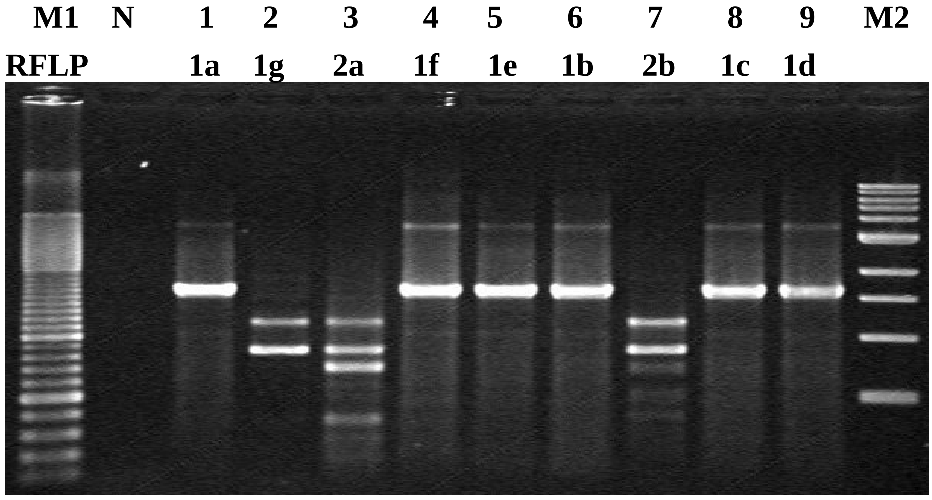

Figure 2. Electrophoretic profile of inverted PCR products of CS-flanking region amplified from plasmids: p2 (lane 1); p11 (lane 2), p13-1 (lane 3), p13-2 (lane 4), p11 (lane 5), p24 (lane 6), p36-1 (lane 7), p36-2 (lane 8), and p35 (lane 9). M1: 100 bp size marker; M2: 1 kb size marker; N: negative control without DNA template; PCR products were separated by 1.0% agarose for 1.5 hr at 50 V and visualized by UV illumination.
