# Supplementary figures and images for: Clonal dissemination of the multi-drug resistant Salmonella enterica serovar Braenderup, but not the serovar Bareilly, of prevalent serogroup C1 Salmonella from Taiwan
Source: BMC Microbiol. 2009 Dec 17;9:264. doi: 10.1186/1471-2180-9-264 (PMC2806260; doi:10.1186/1471-2180-9-264)

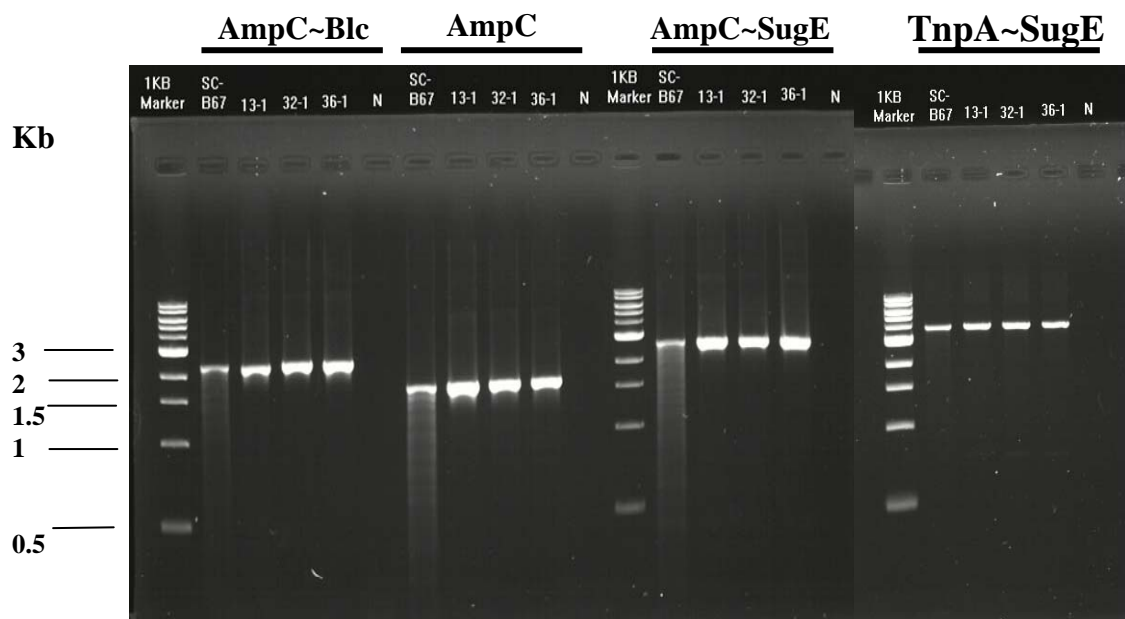

Figure 3. PCR amplification of plasmid-mediated *tnpA-ampC-blc-sugE* genetic structure.

Supplement: Additional file 3 — PCR amplification of plasmid-mediated tnpA-blaCMY-2-blc-sugE genetic structure of type 2 plasmids. All type 2 plasmids consisted of tnpA-blaCMY-2-blc-sugE genetic structure. [file 1471-2180-9-264-S3.PDF]
